# Maternal Bradycardia Associated with Betamethasone Administration During Pregnancy

**DOI:** 10.1155/2019/6873057

**Published:** 2019-10-16

**Authors:** Mojirayo A. Sarumi, James W. Hole, Robert B. Gherman

**Affiliations:** Division of Maternal-Fetal Medicine, WellSpan Health, York, PA, USA

## Abstract

**Background:**

Maternal risks of betamethasone have been rarely reported.

**Case:**

At 36 weeks' gestation, a previously healthy 23-year-old gravida with fetal intrauterine growth restriction was admitted to the hospital for steroid administration. Twenty-six hours after the first dose of betamethasone, a maternal bradycardia was initially noted and eventually nadired at 41 beats per minute. Consultation with the cardio-electrophysiology service revealed no other apparent etiologies for the sinus bradycardia. Due to the asymptomatic nature of the maternal bradycardia, pharmacologic interventions were not recommended. With observation alone, a normal maternal heart rate returned by forty-nine hours after the original betamethasone injection. The patient subsequently had an uneventful intrapartum course.

**Conclusion:**

Maternal bradycardia can be associated with antenatal betamethasone administration. Due to the transient nature of this side effect, expectant management is recommended as the treatment option for asymptomatic patients.

## 1. Introduction

Antenatal corticosteroid administration has been shown to reduce the risk of neonatal intraventricular hemorrhage, necrotizing enterocolitis, and respiratory distress syndrome [1]. Therefore, a single course of corticosteroids is currently recommended for patients at risk of preterm delivery within seven days who are within the gestational age of 24 weeks through 33 weeks and 6 days. Administration of betamethasone to women at risk for late preterm (34 + 0–36 + 6 weeks) delivery has also been shown to be associated with significant reductions in the incidence of severe respiratory complications, transient tachypnea of the newborn, surfactant use, and bronchopulmonary dysplasia [2]. The Antenatal Steroids for Term Elective Caesarean Section trial similarly found lower rates of admission to neonatal intensive care units for respiratory complications in the betamethasone group [3].

Maternal side effects related to betamethasone administration during pregnancy have been rarely reported. Previously reported complications have included maternal hyperglycemia and hypokalemic periodic paralysis [4]. We searched PUBMED, using the mesh terms, “betamethasone,” “bradycardia,” “heart rate” “side effects,” and “pregnancy,” but did not find any previously reported cases of betamethasone associated bradycardia during pregnancy.

## 2. Case

We obtained a waiver from our Institutional Review Board to review this case and patient consent was likewise obtained. A previously healthy 23-year-old Gravida 2 Para 1, presented at 36 + 4 weeks' gestation from an outside hospital for evaluation of severe fetal intra-uterine growth restriction. Her obstetric history was remarkable for an uncomplicated term vaginal delivery. She had no prior history of any cardiac symptoms or diagnosis. The patient denied any exposure to illicit drugs. Her medications included oral prenatal vitamins. Her body mass index was 28.5 kg/m^2^ (182 pounds, k66 inches). She had a normal 1-hour glucose tolerance test of 97 mg/dl. She had received the flu and the tetanus-diphtheria-pertussis vaccines several months prior to her admission. The patient had not received any previous courses of betamethasone during the pregnancy. An initial dose of intramuscular betamethasone had been given eleven hours prior to evaluation at our institution.

**Figure 1:**
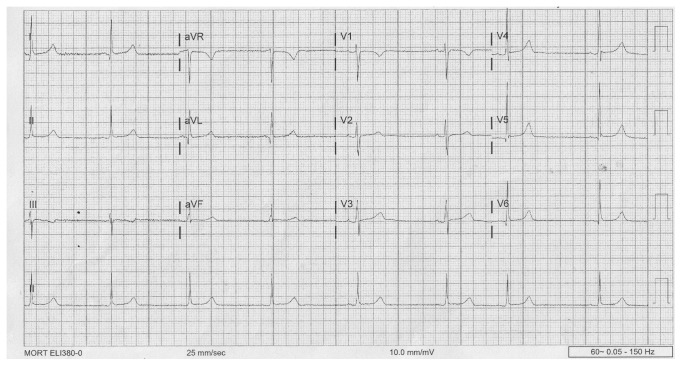
Maternal electrocardiogram from second inpatient day.

The patient's temperature was 97.1 degrees Fahrenheit and her pulse was noted to be 67 beats per minute on admission to our institution. External monitoring showed a reactive fetal heart rate tracing and there were no contractions noted on the tocodynamometer. Ultrasound revealed an estimated fetal weight of 1,704 grams (less than first percentile for gestational age), without any apparent fetal anomalies. Middle cerebral artery (peak systolic velocity 1.29 MoM) and umbilical artery Doppler indices (S/D ratio 2.41, PI 0.83) were both within the normal range. Laboratory evaluation included the following: hematocrit 35.4%, platelets 318 × 10^9^/L, creatinine 0.61 mg/dl, aspartate aminotransferase 11 U/L, alanine aminotransferase 8 U/L, lactate dehydrogenase 111 U/L, uric acid 5.3 mmoL/L, total protein/creatinine ratio 0.14, thyroid stimulating hormone 0.46 mIU/L, and negative urine drug screen. The serum potassium was 4 mmol/L. Throughout the first day of the inpatient admission, the maternal pulse remained between 60 and 72 beats per minute. Two and a half hours after the administration of the second dose of betamethasone acetate, the maternal pulse dropped to below 60 beats per minute (Table 1). On the second inpatient day, the pulse nadired at 41 beats per minute. An electrocardiogram (Figure 1) revealed sinus bradycardia (46 beats per minute, PR 138 s, QRS 86 s, QTC 426 s). The patient remained asymptomatic without any complaints of lightheadedness, dizziness, fatigue, dyspnea, or chest pain. Consultation with the cardio-electrophysiology service revealed no other apparent etiologies for the sinus bradycardia. Due to the asymptomatic nature of the maternal bradycardia, pharmacologic interventions were not recommended. With observation alone, a normal maternal heart rate returned by forty-nine hours after the original betamethasone injection. The patient was discharged to home, with a pulse of 67 beats per minute.

At 36 + 6 weeks' gestation, the patient readmitted and underwent a primary cesarean delivery due to a nonreassuring fetal heart tracing. The patient delivered a 1755-gram viable female infant. Her pulse rate remained within the normal range during the intrapartum and postpartum periods.

## 3. Comment

Sinus bradycardia is defined as a cardiac rate less than sixty beats per minute and occurs when atrial depolarization is initiated from the sino-atrial node. The most common etiology is simply a resting sinus bradycardia. Other etiologies in the differential diagnosis include hypothyroidism, cardiac ischemia or infarction, hypothermia, hyperkalemia, or autonomic disorders that produce an increase in vagal tone relative to sympathetic tone [5].

To date, there have been isolated case reports in the literature of steroid related bradycardia; all of these have occurred in a nonpregnant population. The largest descriptive study to date included sixty-one children treated with one to five mg/kg/day of prednisone [6]. Taylor and Gaco reported a symptomatic sinus bradycardia which was induced by a five-day course of high dose oral prednisolone in a multiple sclerosis patient [7]. Marinov et al. reported a case of sinus bradycardia after a single dose of intravenous dexamethasone for post-operative nausea and vomiting prophylaxis during anesthesia induction [8]. Others have reported sinus bradycardia induced by intravenous methylprednisolone [9, 10].

The precise mechanism through which betamethasone causes bradycardia has not been fully established. Animal studies have shown that high-dose corticosteroid therapy may depress the cardiovascular alpha- and beta-adrenergic receptor sensitivity [11]. Other studies have suggested a transient shift in renal electrolyte excretion [12], steroid-induced variations in blood pressure [10], or abnormal levels of serum electrolytes [13]. Puntis et al. has postulated that the preservatives used in the drug preparation may be etiologic [14].

Our patient had an onset of bradycardia approximately 26 hours after the initial dose of betamethasone. Her bradycardia was noted to resolve approximately 23 hours after its initial diagnosis (49 hours after initial betamethasone administration). This time frame is in keeping with prior reports in the literature, which describe a time range of 48–96 hours of bradycardia onset after initial glucocorticoid administration [6–8]. There have been longer instances of bradycardia onset of 5–7 days after treatment completion [9, 10]. Most of these cases had spontaneous resolution of the bradycardia; medical agents were required in a few instances to ameliorate the bradycardia using intravenous atropine [8], as well as isoproterenol [10].

Our case report possesses limited generalizability, as observational studies with a thorough design and larger patient population are necessary to further evaluate the incidence of betamethasone related maternal bradycardia. Although the package insert for betamethasone describes bradycardia as a potential side effect, we suspect that most obstetricians are not aware of this complication. We propose a consideration of a period of maternal observation and performance of vital signs when corticosteroids are administered in the outpatient setting.

## Figures and Tables

**Table 1:** Vital signs timeline.

	Time	Temperature	Pulse	Respiratory rate	Blood pressure	Oxygen saturation
Hospital admission	01/12/18 21:13:00	37.4 (99.4)	64	16	118/69	95
	01/12/18 22:44:08	36.8 (98.2)	67	14	109/71	97
	01/13/18 05:58:43	36.7 (98.1)	72	14	109/66	95
	01/13/18 07:55:23	36.7 (98.1)	60	20	112/70	95
2 hours, 35 minutes after second dose of BMZ	01/13/18 14:35:45	36.7 (98.1)	59	16	112/59	95
	01/13/18 22:38:50	36.4 (97.6)	*54*	16	117/69	95
	01/14/18 04:50:07	36.7 (98.1)	*49*	*12*	115/77	96
	01/14/18 05:07:21	—	*41*	—	—	95
	01/14/18 07:42:11	36.4 (97.6)	*53*	16	124/81	96
Hospital discharge	01/14/18 13:20:00	36.2 (97.1)	67	16	107/70	96
